# Crystal structures of (*E*)-1-naphthaldehyde oxime and (*E*)-phenanthrene-9-carbaldehyde oxime

**DOI:** 10.1107/S2056989018002116

**Published:** 2018-02-13

**Authors:** Jamal Lasri, Katherine Chulvi, Naser Eltaher Eltayeb

**Affiliations:** a Department of Chemistry, Rabigh College of Science and Arts, PO Box 344, King Abdulaziz University, Jeddah, Saudi Arabia; b FB 1.3 Strukturanalytik, Bundesanstalt für Materialforschung und -prüfung (BAM), Berlin, Germany

**Keywords:** crystal structure, aromatic aldehydes, *E*-aldoximes, hydrogen bonding

## Abstract

The aldoximes C_11_H_9_NO (I) and C_15_H_11_NO (II), synthesized in *ca* 90% yield, by treatment of 1-naphthaldehyde or phenanthrene-9-carbaldehyde, respectively, with hydroxyl­amine hydro­chloride and sodium carbonate, have been characterized by IR, ^1^H, ^13^C and DEPT-135 NMR spectroscopies, and also by single-crystal X-ray diffraction analysis. The mol­ecules of (I) and (II) are conformationally similar, with the aldoxime substituent groups lying outside the planes of the naphthalene or phenanthrene rings, forming dihedral angles with them of 23.9 (4) and 27.9 (6)°, respectively. The crystal structures of both (I) and (II) are similar with a single inter­molecular O—H⋯N hydrogen-bonding inter­action, giving rise to the formation of one-dimensional polymeric chains extending along the 2_1_ (*b*) screw axes in each.

## Abstract 2

The aldoximes (*E*)-1-naphthaldehyde oxime (I) and *E*-phenanthrene-9-carbaldehyde oxime (II) were synthesized and characterized using NMR and XRD. The crystal structures of both (I) and (II) are similar with a single inter­molecular O—H⋯N hydrogen-bonding inter­action, giving rise to the formation of one-dimensional polymeric chains extending along the 2_1_ (*b*) screw axes in each.

## Chemical context   

Oxime compounds have found many applications; for example in the medical field, they are used as anti­dotes for nerve agents (Kassa, 2002 ▸). Oximes are also used as inter­mediates in the industrial production of caprolactam, a precursor to Nylon 6 (Ritz *et al.*, 2012 ▸). Oximes, HO—N=C*R* ^1^ *R* ^2^, are also valuable and simple reagents containing the O—N=C moiety (Kukushkin & Pombeiro, 1999 ▸), which easily adds to nitrile ligands, to form a variety of nitro­gen-containing products *e.g.* imino­acyl­ated compounds (Kopylovich *et al.*, 2009 ▸; Lasri *et al.*, 2007 ▸, 2008 ▸), amidines (Kopylovich *et al.*, 2001 ▸), carboxamides (Kopylovich *et al.*, 2002 ▸), phthalocyanines (Kopylovich *et al.*, 2004 ▸), or 1,3,5-tri­aza­penta­diene species (Kopylovich *et al.*, 2007 ▸). In this work, we report the synthesis and crystal structures of two aldoximes, *viz.* (*E*)-1-naphthaldehyde oxime (I) and (*E*)-phenanthrene-9-carbaldehyde oxime (II), by treatment of 1-naphthaldehyde or phenanthrene-9-carbaldehyde, respectively, with hydroxyl­amine hydro­chloride and sodium carbonate.
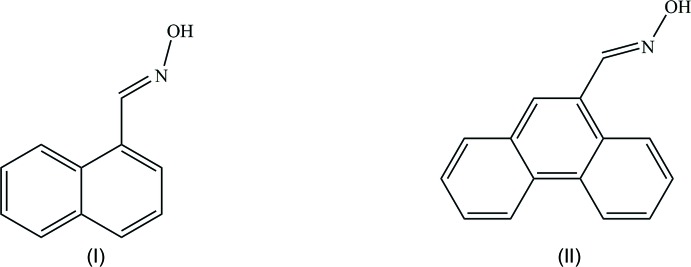


## Structural commentary   

**Figure 1:**
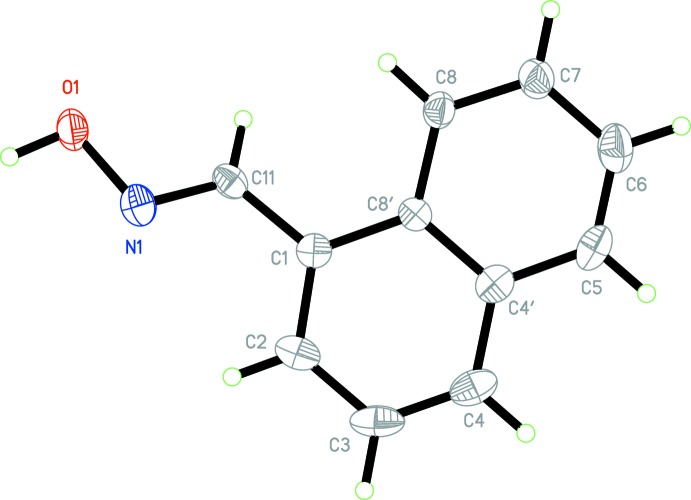
The mol­ecular conformation and atom-numbering scheme for (I), with non-H atoms represented as 30% probability ellipsoids.

The title compounds (I) and (II) both crystallize in the non-centrosymmetric monoclinic space group *P*2_1_ with *Z* = 2, with similar unit-cell parameters. The asymmetric unit contents for each are shown in Figs. 1 ▸ and 2 ▸. Compound (I) comprises a naphthalene unit functionalized with an aldoxime group at position 1. The naphthalene unit is, as expected, essentially planar but the plane containing the aldoxime atoms lies significantly out of the naphthalene plane [torsion angle N1—C11—C1—C2 = 23.6 (6)°] (Table 1 ▸). In the case of compound (II), the plane of the aldoxime group lies similarly out-of-plane with the phenanthrene ring system [comparative torsion angle N1—C11—C9—C10 = 27.6 (4)°], corresponding to dihedral angles between the two planes of 23.9 (4) and 27.9 (5)° for (I) and (II), respectively. The aldoxime group shows similar bond lengths for both structures: 1.395 (5) and 1.405 (3) Å for O1—N1, 1.273 (5) and 1.268 (3) Å for N1—C11, 1.461 (6) and 1.466 (4) Å for C1—C11 or C9—C11, for (I) and (II), respectively.

## Supra­molecular features   

**Figure 3:**
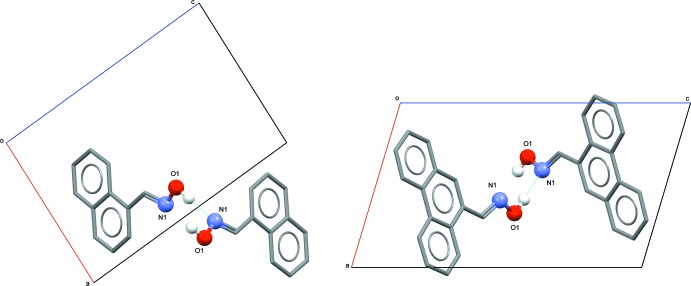
Inter­molecular hydrogen-bonding associations for (I) (left) and (II) (right), shown as dashed lines. Non-associative H atoms have been omitted for clarity.

**Figure 4:**
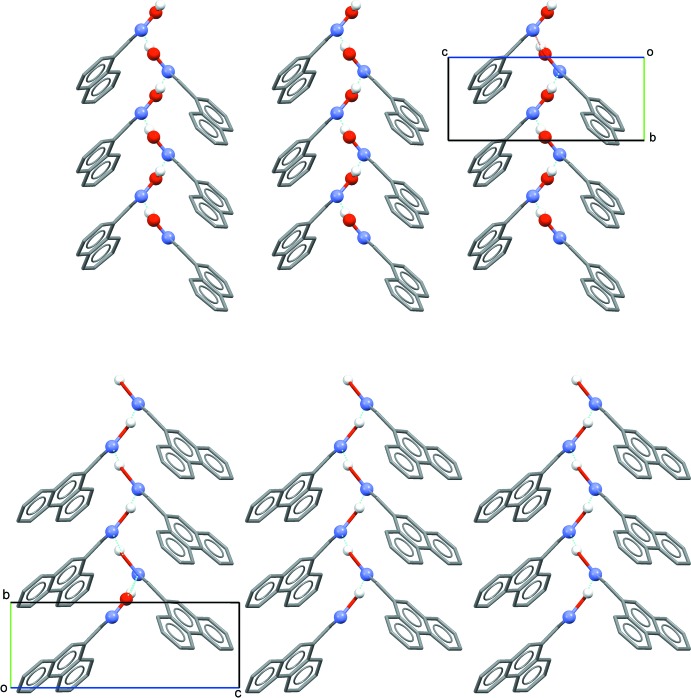
A packing diagram viewed along the *a* axis for (I) (top) and (II) (bottom), showing polymeric chain extensions.

Similar inter­molecular inter­actions are observed in the crystal structures of both (I) and (II). In each, mol­ecules are linked through a single inter­molecular O1—H⋯N1^i^ hydrogen-bonding inter­action [Tables 2 ▸ and 3 ▸ for (I) and (II), respectively]. These basic inter­actions are shown in Fig. 3 ▸, defining an oxime *C*(3) type II motif. It is well known that oximes are able to form different types of hydrogen-bonding motifs (Bruton *et al.*, 2003 ▸). In the structures of both (I) and (II), the formation of a one-dimensional polymeric chain arrangement of mol­ecules results, extending along the 2_1_ (*b*) screw axes in each (Fig. 4 ▸).

## Database survey   

Many naphthalene-carbaldehyde oxime derivatives are present in the Cambridge Structural Database (Version 5.38; Groom *et al.* 2016 ▸) but no one crystal structure containing only an aldoxime group in position 1 of the naphthalene ring system has been reported. The most similar structures that can be found are LIVROY/LIVROY01 (Guo *et al.*, 2008 ▸; Tarai & Baruah, 2016 ▸) with an additional hydroxyl group in position 2 and TIJPOS (Asaad *et al.*, 2005 ▸) with a di­methyl­amino group in position 9. The most important difference between (I) and LIVROY/LIVROY01 are the two hydrogen bonds: one intra­molecular O—H⋯N and another inter­molecular O—H⋯O. As a result of the intra­molecular hydrogen-bonding inter­action, the aldoxime group in the latter compound is coplanar with the central naphthalene ring with a dihedral angle of 1.21° and torsion angles C1—C11—N1—O2 = 179.27, C3—C1—C11—N1 = −179.91 and C4—C1—C11—N1 = −0.76°. However, TIJPOS (Asaad *et al.*, 2005 ▸), with just one type of inter­molecular hydrogen bond, shows a rotation in the aldoxime group that is more dramatic than in (I) and (II) (Table 1 ▸), with a 40.35° deviation from the central naphthalene plane.

No examples of structures of phenanthrene-carbaldehyde oxime derivatives are present in the Cambridge Structural Database.

## Synthesis and crystallization   

The aldoximes (*E*)-1-naphthaldehyde oxime (I) and (*E*)-phen­an­threne-9-carbaldehyde oxime (II) were synthesized, in *ca* 90% yield, by treatment of 1-naphthaldehyde or phenanthrene-9-carbaldehyde, respectively, with hydroxyl­amine hydro­chloride and sodium carbonate in MeOH at room temperature. To a solution of hydroxyl­amine hydro­chloride (41.6 mg, 0.60 mmol) in MeOH (10 ml) was added sodium carbonate (31.7 mg, 0.30 mmol). The reaction mixture was stirred at room temperature for 5 min. 1-Naphthaldehyde (85.0 mg, 0.54 mmol) or phenanthrene-9-carbaldehyde (112.2 mg, 0.54 mmol) was added and the reaction mixture was stirred at room temperature for 12 h. The precipitate formed was then filtered off and the filtrate was evaporated *in vacuo*. The crude residue was purified by column chroma­tography on silica (CHCl_3_ as the eluent, 50 ml), followed by evaporation of the solvent *in vacuo* to give the pure aldoximes [(I), 84 mg, 90% yield and (II), 107 mg, 89% yield] (see reaction scheme).
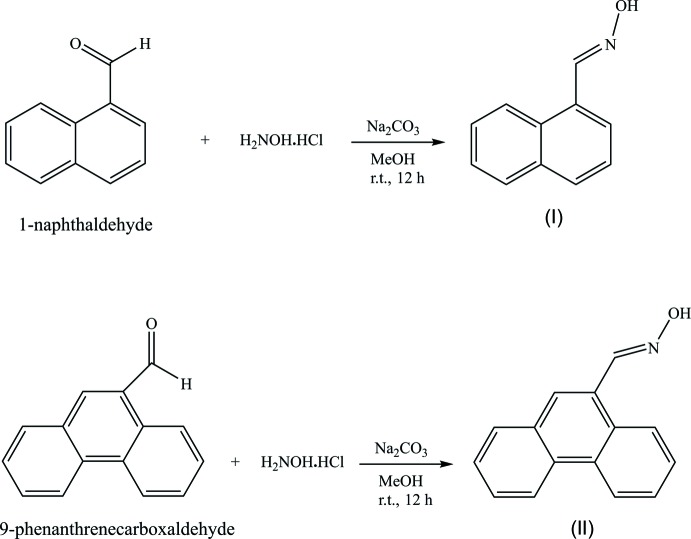


Single crystals of the aldoximes (I) and (II) suitable for X-ray diffraction were obtained by slow evaporation of their 10 ml CHCl_3_ solutions at room temperature. Compounds (I) and (II) were characterized by IR, ^1^H, ^13^C and DEPT-135 NMR spectroscopies and also by single crystal X-ray diffraction analysis.

In the IR spectra of (I) and (II), the characteristic bands at wavenumbers 3389 and 3200 cm^−1^ (O—H), and 1614 and 1607 cm-^1^ (C=N), confirm the formation of the aldoximes (I) and (II), respectively. In the ^1^H NMR spectra, we observed the absence of the signal of the aldehyde at *ca* 10 ppm and a new signal at *ca* 8.8 ppm due to the imine proton C*H*=N was detected. Moreover, in the ^13^C and DEPT-135 NMR spectra, the signal of the aldehyde at *ca* 190 ppm was not observed, and a new signal at *ca* 150 ppm due to the oxime carbon *C*H=NOH was detected, confirming the formation of the aldoximes (I) and (II).

**(** ***E*** **)-1-naphthaldehyde oxime (I)**

Yield: 90%. IR (cm^−1^): 3389 (OH), 1614 (C=N). ^1^H NMR (CDCl_3_), *δ*: 7.53 (*t*, *J*_HH_ 7.5 Hz, 1H, C*H*_aromatic_), 7.56 (*t*, *J*_HH_ 7.0 Hz, 1H, C*H*_aromatic_), 7.61 (*t*, *J*_HH_ 7.0 Hz, 1H, C*H*_aromatic_), 7.82 (*d*, *J*_HH_ 7.1 Hz, 1H, C*H*_aromatic_), 7.93 (*t*, *J*_HH_ 8.1 Hz, 2H, C*H*_aromatic_), 8.48 (*d*, *J*_HH_ 8.3 Hz, 1H, C*H*_aromatic_), 8.87 (*s*, 1H, C*H*=N). ^13^C NMR (CDCl_3_), *δ*: 124.2, 125.4, 126.2, 127.0, 127.1 (*C*H_aromatic_), 128.0 (C_aromatic_), 128.8, 130.6 (*C*H_aromatic_), 130.8, 133.8 (C_aromatic_), 150.0 (*C*H=N). DEPT-135 NMR (CDCl_3_), *δ*: 124.2, 125.4, 126.2, 127.0, 127.1, 128.8, 130.6 (*C*H_aromatic_), 150.0 (*C*H=N).

***E*** **-phenanthrene-9-carbaldehyde oxime (II)**

Yield: 89%. IR (cm^−1^): 3200 (OH), 1607 (C=N). ^1^H NMR (CDCl_3_), *δ*: 7.64 (*t*, *J*_HH_ 7.9 Hz, 1H, C*H*_aromatic_), 7.68–7.75 (m, 3H, C*H*_aromatic_), 7.94 (*d*, *J*_HH_ 7.9 Hz, 1H, C*H*_aromatic_), 8.04 (*s*, 1H, C*H*_aromatic_), 8.62 (*d*, *J*_HH_ 7.9 Hz, 1H, C*H*_aromatic_), 8.70 (*d*, *J*_HH_ 8.2 Hz, 1H, C*H*_aromatic_), 8.77 (*d*, *J*_HH_ 8.2 Hz, 1H, C*H*_aromatic_), 8.85 (*s*, 1H, C*H*=N). ^13^C NMR (CDCl_3_), *δ*: 122.6, 123.1, 125.4 (*C*H_aromatic_), 126.8 (C_aromatic_), 126.9, 127.0, 127.2, 127.9, 129.3 (*C*H_aromatic_), 130.7, 131.0, 131.1 (C_aromatic_), 150.8 (*C*H=N). DEPT-135 NMR (CDCl_3_), *δ*: 122.6, 123.1, 125.4, 126.9, 127.0, 127.2, 127.9, 129.3 (*C*H_aromatic_), 150.8 (*C*H=N).

## Refinement   

Crystal data, data collection and structure refinement details are summarized in Table 4 ▸. All C-bound H atoms were located in difference-Fourier maps but were subsequently treated as riding with C—H = 0.93 Å and with *U*_iso_(H) = 1.2*U*_eq_(C). The H atoms of the OH groups were positioned with idealized geometry and were refined freely in both structures.

## Supplementary Material

Crystal structure: contains datablock(s) I, II, global. DOI: 10.1107/S2056989018002116/zs2397sup1.cif

Structure factors: contains datablock(s) I. DOI: 10.1107/S2056989018002116/zs2397Isup4.hkl

Structure factors: contains datablock(s) II. DOI: 10.1107/S2056989018002116/zs2397IIsup5.hkl

Supporting information file. DOI: 10.1107/S2056989018002116/zs2397Isup4.cml

Supporting information file. DOI: 10.1107/S2056989018002116/zs2397IIsup5.cml

CCDC references: 1821856, 1821855

Additional supporting information:  crystallographic information; 3D view; checkCIF report

## Figures and Tables

**Figure 2:**
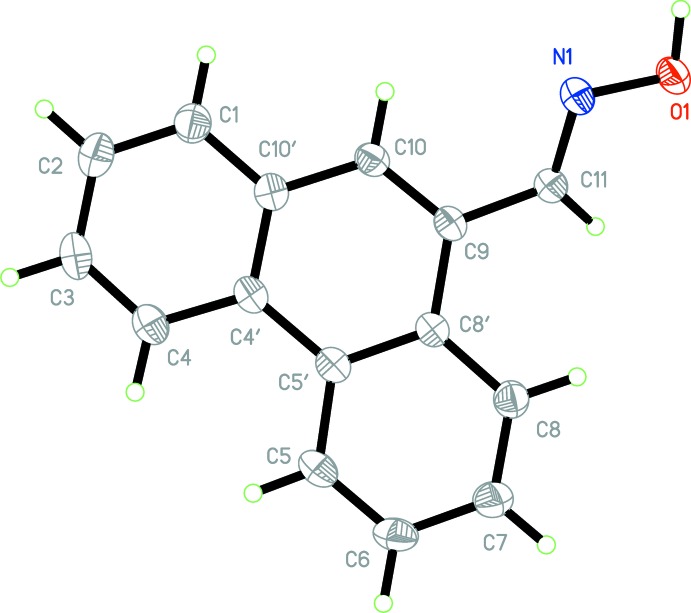
The mol­ecular conformation and atom-numbering scheme for (II), with non-H atoms represented as 30% probability ellipsoids.

**Table 1:** Selected torsion angles (°) for the aldoxime groups in (I) and (II)

	Compound (I)	Compound (II)
C1/C9—C11—N1—O1	−175.5 (4)	−175.3 (2)
C2/C10—C1/C9—C11—N1	23.6 (6)	27.6 (4)
C8′—C1—C11—N1	−160.4 (4)	–
C8′—C9—C11—N1	–	−156.1 (2)

**Table 2:** Hydrogen-bond geometry (Å, °) for (I)

*D*—H⋯*A*	*D*—H	H⋯*A*	*D*⋯*A*	*D*—H⋯*A*
O1—H1⋯N1^i^	0.90 (6)	1.94 (6)	2.834 (5)	177 (6)

Symmetry code: (i) 
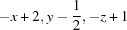
.

**Table 3:** Hydrogen-bond geometry (Å, °) for (II)

*D*—H⋯*A*	*D*—H	H⋯*A*	*D*⋯*A*	*D*—H⋯*A*
O1—H⋯N1^i^	0.88 (3)	1.99 (3)	2.852 (3)	169 (3)

Symmetry code: (i) 
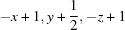
.

**Table 4:** Experimental details

	(I)	(II)
Crystal data		
Chemical formula	C_11_H_9_NO	C_15_H_11_NO
*M*_r_	171.19	221.25
Crystal system, space group	Monoclinic, *P*2_1_	Monoclinic, *P*2_1_
Temperature (K)	295	295
*a*, *b*, *c* (Å)	7.928 (5), 4.843 (3), 11.444 (7)	8.2397 (8), 4.9728 (5), 13.9332 (14)
β (°)	94.03 (5)	106.680 (7)
*V* (Å^3^)	438.3 (5)	546.88 (10)
*Z*	2	2
Radiation type	Mo *K*α	Mo *K*α
μ (mm^−1^)	0.08	0.09
Crystal size (mm)	0.10 × 0.06 × 0.02	0.16 × 0.09 × 0.05

Data collection		
Diffractometer	Bruker D8 Quest	Bruker D8 Quest
Absorption correction	Multi-scan (*SADABS*; Bruker, 2016 ▸)	Multi-scan (*SADABS*; Bruker, 2016 ▸)
*T*_min_, *T*_max_	0.684, 0.745	0.698, 0.745
No. of measured, independent and observed [*I* > 2σ(*I*)] reflections	5762, 1570, 957	7330, 1988, 1509
*R*_int_	0.100	0.053
(sin θ/λ)_max_ (Å^−1^)	0.603	0.602

Refinement		
*R*[*F* ^2^ > 2σ(*F* ^2^)], *wR*(*F* ^2^), *S*	0.055, 0.098, 1.06	0.040, 0.093, 1.04
No. of reflections	1570	1988
No. of parameters	122	159
No. of restraints	1	1
H-atom treatment	H atoms treated by a mixture of independent and constrained refinement	H atoms treated by a mixture of independent and constrained refinement
Δρ_max_, Δρ_min_ (e Å^−3^)	0.15, −0.15	0.15, −0.15

Computer programs: *APEX3* and *SAINT* (Bruker, 2016 ▸), *SHELXT2014* (Sheldrick, 2015*a* ▸) and *SHELXL2014* (Sheldrick, 2015*b* ▸).

## supplementary crystallographic information

### (E)-1-Naphthaldehyde oxime (I) Crystal data

**Table:**

C_11_H_9_NO	*F*(000) = 180
*M**_r_* = 171.19	*D*_x_ = 1.297 Mg m^−^^3^
Monoclinic, *P*2_1_	Mo *K*α radiation, λ = 0.71073 Å
*a* = 7.928 (5) Å	Cell parameters from 1367 reflections
*b* = 4.843 (3) Å	θ = 2.6–22.6°
*c* = 11.444 (7) Å	µ = 0.08 mm^−^^1^
β = 94.03 (5)°	*T* = 295 K
*V* = 438.3 (5) Å^3^	Block, colourless
*Z* = 2	0.10 × 0.06 × 0.02 mm

### (E)-1-Naphthaldehyde oxime (I) Data collection

**Table:**

Bruker D8 Quest diffractometer	957 reflections with *I* > 2σ(*I*)
φ and ω scans	*R*_int_ = 0.100
Absorption correction: multi-scan (SADABS; Bruker, 2016)	θ_max_ = 25.4°, θ_min_ = 2.6°
*T*_min_ = 0.684, *T*_max_ = 0.745	*h* = −9→9
5762 measured reflections	*k* = −5→5
1570 independent reflections	*l* = −13→13

### (E)-1-Naphthaldehyde oxime (I) Refinement

**Table:**

Refinement on *F*^2^	1 restraint
Least-squares matrix: full	Hydrogen site location: mixed
*R*[*F*^2^ > 2σ(*F*^2^)] = 0.055	H atoms treated by a mixture of independent and constrained refinement
*wR*(*F*^2^) = 0.098	*w* = 1/[σ^2^(*F*_o_^2^) + (0.0164*P*)^2^ + 0.1408*P*] where *P* = (*F*_o_^2^ + 2*F*_c_^2^)/3
*S* = 1.06	(Δ/σ)_max_ < 0.001
1570 reflections	Δρ_max_ = 0.15 e Å^−^^3^
122 parameters	Δρ_min_ = −0.15 e Å^−^^3^

### (E)-1-Naphthaldehyde oxime (I) Special details

**Table:**

Geometry. All esds (except the esd in the dihedral angle between two l.s. planes) are estimated using the full covariance matrix. The cell esds are taken into account individually in the estimation of esds in distances, angles and torsion angles; correlations between esds in cell parameters are only used when they are defined by crystal symmetry. An approximate (isotropic) treatment of cell esds is used for estimating esds involving l.s. planes.

### (E)-1-Naphthaldehyde oxime (I) Fractional atomic coordinates and isotropic or equivalent isotropic displacement parameters (Å^2^)

**Table:**

	*x*	*y*	*z*	*U*_iso_*/*U*_eq_	
O1	0.8254 (4)	−0.0401 (7)	0.5056 (3)	0.0458 (9)	
N1	0.8708 (5)	0.1715 (9)	0.4312 (3)	0.0418 (11)	
C1	0.7559 (5)	0.4959 (10)	0.2895 (4)	0.0318 (12)	
C2	0.8998 (6)	0.5156 (11)	0.2295 (4)	0.0452 (14)	
H2	0.9903	0.3987	0.2494	0.054*	
C3	0.9129 (7)	0.7072 (14)	0.1393 (5)	0.0569 (16)	
H3	1.0128	0.7207	0.1017	0.068*	
C4	0.7801 (7)	0.8737 (11)	0.1066 (4)	0.0499 (15)	
H4	0.7888	0.9970	0.0450	0.060*	
C4'	0.6296 (6)	0.8624 (10)	0.1645 (4)	0.0377 (12)	
C5	0.4924 (6)	1.0383 (10)	0.1338 (4)	0.0468 (15)	
H5	0.5009	1.1626	0.0726	0.056*	
C6	0.3485 (7)	1.0323 (12)	0.1907 (5)	0.0570 (17)	
H6	0.2585	1.1479	0.1677	0.068*	
C7	0.3370 (6)	0.8491 (11)	0.2846 (4)	0.0478 (15)	
H7	0.2393	0.8461	0.3250	0.057*	
C8	0.4663 (5)	0.6760 (11)	0.3176 (4)	0.0387 (13)	
H8	0.4557	0.5558	0.3800	0.046*	
C8'	0.6172 (5)	0.6760 (10)	0.2582 (4)	0.0285 (11)	
C11	0.7413 (6)	0.2826 (9)	0.3785 (4)	0.0350 (12)	
H11	0.6343	0.2264	0.3972	0.042*	
H1	0.922 (7)	−0.129 (13)	0.528 (5)	0.10 (2)*	

### (E)-1-Naphthaldehyde oxime (I) Atomic displacement parameters (Å^2^)

**Table:**

	*U*^11^	*U*^22^	*U*^33^	*U*^12^	*U*^13^	*U*^23^
O1	0.041 (2)	0.044 (2)	0.051 (2)	0.008 (2)	−0.0047 (18)	0.008 (2)
N1	0.040 (2)	0.038 (3)	0.046 (3)	0.001 (2)	−0.005 (2)	−0.005 (2)
C1	0.033 (3)	0.029 (3)	0.033 (3)	−0.007 (3)	−0.002 (2)	−0.007 (3)
C2	0.035 (3)	0.054 (4)	0.048 (3)	−0.005 (3)	0.009 (2)	−0.014 (3)
C3	0.051 (3)	0.080 (5)	0.043 (3)	−0.018 (4)	0.021 (3)	−0.012 (4)
C4	0.064 (4)	0.051 (4)	0.036 (3)	−0.017 (3)	0.011 (3)	−0.005 (3)
C4'	0.046 (3)	0.037 (3)	0.029 (3)	−0.008 (3)	−0.003 (2)	−0.007 (3)
C5	0.060 (4)	0.037 (4)	0.041 (3)	−0.009 (3)	−0.011 (3)	0.008 (3)
C6	0.049 (3)	0.049 (4)	0.070 (4)	0.006 (3)	−0.013 (3)	0.008 (4)
C7	0.035 (3)	0.048 (4)	0.060 (4)	0.002 (3)	0.000 (3)	0.009 (3)
C8	0.036 (3)	0.039 (3)	0.040 (3)	0.002 (3)	−0.002 (2)	0.008 (3)
C8'	0.029 (2)	0.027 (3)	0.029 (3)	−0.004 (2)	−0.0016 (19)	−0.008 (3)
C11	0.032 (3)	0.029 (3)	0.044 (3)	0.002 (2)	0.003 (2)	−0.008 (3)

### (E)-1-Naphthaldehyde oxime (I) Geometric parameters (Å, º)

**Table:**

O1—N1	1.395 (5)	C4'—C5	1.407 (6)
O1—H1	0.90 (6)	C4'—C8'	1.410 (6)
N1—C11	1.273 (5)	C5—C6	1.353 (6)
C1—C2	1.375 (6)	C5—H5	0.9300
C1—C8'	1.429 (6)	C6—C7	1.402 (7)
C1—C11	1.461 (6)	C6—H6	0.9300
C2—C3	1.397 (7)	C7—C8	1.357 (6)
C2—H2	0.9300	C7—H7	0.9300
C3—C4	1.358 (7)	C8—C8'	1.417 (5)
C3—H3	0.9300	C8—H8	0.9300
C4—C4'	1.406 (6)	C11—H11	0.9300
C4—H4	0.9300		
			
N1—O1—H1	106 (4)	C6—C5—H5	119.0
C11—N1—O1	111.5 (4)	C4'—C5—H5	119.0
C2—C1—C8'	118.9 (4)	C5—C6—C7	119.0 (5)
C2—C1—C11	120.4 (5)	C5—C6—H6	120.5
C8'—C1—C11	120.6 (4)	C7—C6—H6	120.5
C1—C2—C3	121.5 (5)	C8—C7—C6	121.0 (5)
C1—C2—H2	119.3	C8—C7—H7	119.5
C3—C2—H2	119.3	C6—C7—H7	119.5
C4—C3—C2	120.1 (5)	C7—C8—C8'	120.9 (5)
C4—C3—H3	119.9	C7—C8—H8	119.5
C2—C3—H3	119.9	C8'—C8—H8	119.5
C3—C4—C4'	120.9 (5)	C4'—C8'—C8	118.1 (4)
C3—C4—H4	119.5	C4'—C8'—C1	119.2 (4)
C4'—C4—H4	119.5	C8—C8'—C1	122.7 (4)
C4—C4'—C5	121.7 (5)	N1—C11—C1	121.9 (4)
C4—C4'—C8'	119.3 (5)	N1—C11—H11	119.0
C5—C4'—C8'	118.9 (4)	C1—C11—H11	119.0
C6—C5—C4'	122.0 (5)		
			
C8'—C1—C2—C3	0.1 (7)	C5—C4'—C8'—C8	−0.3 (6)
C11—C1—C2—C3	176.2 (5)	C4—C4'—C8'—C1	−2.1 (6)
C1—C2—C3—C4	−2.0 (8)	C5—C4'—C8'—C1	179.7 (4)
C2—C3—C4—C4'	1.8 (8)	C7—C8—C8'—C4'	0.5 (7)
C3—C4—C4'—C5	178.3 (5)	C7—C8—C8'—C1	−179.6 (4)
C3—C4—C4'—C8'	0.3 (7)	C2—C1—C8'—C4'	1.9 (6)
C4—C4'—C5—C6	−178.7 (5)	C11—C1—C8'—C4'	−174.2 (4)
C8'—C4'—C5—C6	−0.6 (7)	C2—C1—C8'—C8	−178.0 (4)
C4'—C5—C6—C7	1.3 (8)	C11—C1—C8'—C8	5.9 (6)
C5—C6—C7—C8	−1.2 (8)	O1—N1—C11—C1	−175.5 (4)
C6—C7—C8—C8'	0.2 (7)	C2—C1—C11—N1	23.6 (6)
C4—C4'—C8'—C8	177.8 (4)	C8'—C1—C11—N1	−160.4 (4)

### (E)-1-Naphthaldehyde oxime (I) Hydrogen-bond geometry (Å, º)

**Table:**

*D*—H···*A*	*D*—H	H···*A*	*D*···*A*	*D*—H···*A*
O1—H1···N1^i^	0.90 (6)	1.94 (6)	2.834 (5)	177 (6)

Symmetry code: (i) −*x*+2, *y*−1/2, −*z*+1.

### (E)-Phenanthrene-9-carbaldehyde oxime (II) Crystal data

**Table:**

C_15_H_11_NO	*F*(000) = 232
*M**_r_* = 221.25	*D*_x_ = 1.344 Mg m^−^^3^
Monoclinic, *P*2_1_	Mo *K*α radiation, λ = 0.71073 Å
*a* = 8.2397 (8) Å	Cell parameters from 2141 reflections
*b* = 4.9728 (5) Å	θ = 2.6–24.9°
*c* = 13.9332 (14) Å	µ = 0.09 mm^−^^1^
β = 106.680 (7)°	*T* = 295 K
*V* = 546.88 (10) Å^3^	Block, colourless
*Z* = 2	0.16 × 0.09 × 0.05 mm

### (E)-Phenanthrene-9-carbaldehyde oxime (II) Data collection

**Table:**

Bruker D8 Quest diffractometer	1509 reflections with *I* > 2σ(*I*)
φ and ω scans	*R*_int_ = 0.053
Absorption correction: multi-scan (SADABS; Bruker, 2016)	θ_max_ = 25.3°, θ_min_ = 2.6°
*T*_min_ = 0.698, *T*_max_ = 0.745	*h* = −9→9
7330 measured reflections	*k* = −5→5
1988 independent reflections	*l* = −16→16

### (E)-Phenanthrene-9-carbaldehyde oxime (II) Refinement

**Table:**

Refinement on *F*^2^	Hydrogen site location: mixed
Least-squares matrix: full	H atoms treated by a mixture of independent and constrained refinement
*R*[*F*^2^ > 2σ(*F*^2^)] = 0.040	*w* = 1/[σ^2^(*F*_o_^2^) + (0.0478*P*)^2^] where *P* = (*F*_o_^2^ + 2*F*_c_^2^)/3
*wR*(*F*^2^) = 0.093	(Δ/σ)_max_ < 0.001
*S* = 1.04	Δρ_max_ = 0.15 e Å^−^^3^
1988 reflections	Δρ_min_ = −0.15 e Å^−^^3^
159 parameters	Extinction correction: SHELXL2016 (Sheldrick, 2015b), Fc^*^=kFc[1+0.001xFc^2^λ^3^/sin(2θ)]^-1/4^
1 restraint	Extinction coefficient: 0.058 (11)

### (E)-Phenanthrene-9-carbaldehyde oxime (II) Special details

**Table:**

Geometry. All esds (except the esd in the dihedral angle between two l.s. planes) are estimated using the full covariance matrix. The cell esds are taken into account individually in the estimation of esds in distances, angles and torsion angles; correlations between esds in cell parameters are only used when they are defined by crystal symmetry. An approximate (isotropic) treatment of cell esds is used for estimating esds involving l.s. planes.

### (E)-Phenanthrene-9-carbaldehyde oxime (II) Fractional atomic coordinates and isotropic or equivalent isotropic displacement parameters (Å^2^)

**Table:**

	*x*	*y*	*z*	*U*_iso_*/*U*_eq_	
O1	0.3294 (3)	1.0407 (5)	0.49418 (15)	0.0446 (6)	
N1	0.4084 (3)	0.8293 (5)	0.55737 (16)	0.0371 (6)	
C11	0.3086 (4)	0.7264 (5)	0.6016 (2)	0.0340 (7)	
H11	0.197024	0.785637	0.585614	0.041*	
C9	0.3660 (3)	0.5157 (6)	0.67732 (18)	0.0322 (7)	
C10	0.5314 (4)	0.5027 (7)	0.7316 (2)	0.0366 (7)	
H10	0.607395	0.622514	0.716727	0.044*	
C10'	0.5935 (3)	0.3140 (6)	0.81023 (19)	0.0349 (7)	
C1	0.7655 (4)	0.3096 (7)	0.8651 (2)	0.0472 (8)	
H1	0.840812	0.427685	0.848577	0.057*	
C2	0.8232 (4)	0.1335 (8)	0.9426 (2)	0.0510 (9)	
H2	0.937339	0.131717	0.978623	0.061*	
C3	0.7116 (4)	−0.0423 (7)	0.9676 (2)	0.0481 (9)	
H3	0.751583	−0.162219	1.020293	0.058*	
C4	0.5438 (4)	−0.0424 (6)	0.9159 (2)	0.0445 (8)	
H4	0.470941	−0.162230	0.933886	0.053*	
C4'	0.4792 (4)	0.1366 (6)	0.83561 (19)	0.0333 (7)	
C5'	0.3013 (4)	0.1442 (6)	0.77844 (19)	0.0334 (7)	
C5	0.1811 (4)	−0.0332 (6)	0.7975 (2)	0.0431 (8)	
H5	0.215809	−0.156417	0.849626	0.052*	
C6	0.0153 (4)	−0.0300 (7)	0.7419 (3)	0.0499 (9)	
H6	−0.061247	−0.149898	0.756119	0.060*	
C7	−0.0389 (4)	0.1524 (7)	0.6641 (2)	0.0499 (9)	
H7	−0.151904	0.154288	0.626056	0.060*	
C8	0.0728 (4)	0.3292 (7)	0.6429 (2)	0.0424 (8)	
H8	0.034520	0.451063	0.590667	0.051*	
C8'	0.2453 (3)	0.3306 (6)	0.69893 (19)	0.0317 (7)	
H	0.413 (4)	1.109 (7)	0.475 (2)	0.061 (12)*	

### (E)-Phenanthrene-9-carbaldehyde oxime (II) Atomic displacement parameters (Å^2^)

**Table:**

	*U*^11^	*U*^22^	*U*^33^	*U*^12^	*U*^13^	*U*^23^
O1	0.0474 (13)	0.0426 (14)	0.0448 (12)	0.0035 (11)	0.0145 (10)	0.0153 (11)
N1	0.0446 (14)	0.0344 (14)	0.0343 (13)	0.0040 (13)	0.0142 (11)	0.0020 (12)
C11	0.0375 (16)	0.0301 (17)	0.0356 (15)	0.0031 (14)	0.0127 (13)	−0.0001 (13)
C9	0.0408 (16)	0.0295 (16)	0.0284 (14)	0.0016 (16)	0.0131 (12)	−0.0015 (14)
C10	0.0416 (16)	0.0334 (17)	0.0352 (14)	−0.0037 (15)	0.0116 (12)	0.0008 (15)
C10'	0.0427 (16)	0.0307 (16)	0.0313 (14)	0.0026 (15)	0.0106 (12)	−0.0056 (14)
C1	0.0453 (18)	0.046 (2)	0.0475 (18)	0.0005 (19)	0.0093 (14)	0.0020 (17)
C2	0.0461 (19)	0.053 (2)	0.0475 (19)	0.0078 (18)	0.0024 (15)	−0.0014 (17)
C3	0.060 (2)	0.044 (2)	0.0371 (17)	0.0147 (18)	0.0089 (15)	0.0060 (15)
C4	0.0555 (19)	0.041 (2)	0.0390 (17)	0.0035 (17)	0.0168 (14)	0.0040 (15)
C4'	0.0464 (17)	0.0265 (15)	0.0294 (14)	0.0033 (15)	0.0149 (13)	−0.0035 (13)
C5'	0.0432 (16)	0.0297 (15)	0.0306 (14)	0.0021 (15)	0.0157 (12)	−0.0040 (14)
C5	0.0552 (19)	0.036 (2)	0.0441 (18)	−0.0002 (17)	0.0247 (15)	0.0039 (15)
C6	0.0458 (19)	0.047 (2)	0.065 (2)	−0.0071 (18)	0.0282 (16)	0.0019 (18)
C7	0.0401 (18)	0.051 (2)	0.059 (2)	−0.0031 (18)	0.0148 (15)	0.0046 (19)
C8	0.0440 (18)	0.0402 (18)	0.0413 (17)	0.0011 (17)	0.0096 (14)	0.0018 (16)
C8'	0.0386 (15)	0.0285 (15)	0.0305 (14)	0.0027 (15)	0.0139 (12)	−0.0031 (14)

### (E)-Phenanthrene-9-carbaldehyde oxime (II) Geometric parameters (Å, º)

**Table:**

O1—N1	1.405 (3)	C3—C4	1.363 (4)
O1—H	0.88 (3)	C3—H3	0.9300
N1—C11	1.268 (3)	C4—C4'	1.409 (4)
C11—C9	1.466 (4)	C4—H4	0.9300
C11—H11	0.9300	C4'—C5'	1.454 (4)
C9—C10	1.357 (3)	C5'—C5	1.408 (4)
C9—C8'	1.448 (4)	C5'—C8'	1.416 (4)
C10—C10'	1.422 (4)	C5—C6	1.364 (4)
C10—H10	0.9300	C5—H5	0.9300
C10'—C1	1.405 (4)	C6—C7	1.385 (4)
C10'—C4'	1.408 (4)	C6—H6	0.9300
C1—C2	1.365 (4)	C7—C8	1.365 (4)
C1—H1	0.9300	C7—H7	0.9300
C2—C3	1.384 (5)	C8—C8'	1.412 (4)
C2—H2	0.9300	C8—H8	0.9300
			
N1—O1—H	102 (2)	C3—C4—H4	119.5
C11—N1—O1	110.9 (2)	C4'—C4—H4	119.5
N1—C11—C9	121.2 (3)	C10'—C4'—C4	117.9 (2)
N1—C11—H11	119.4	C10'—C4'—C5'	119.2 (2)
C9—C11—H11	119.4	C4—C4'—C5'	122.9 (3)
C10—C9—C8'	119.6 (2)	C5—C5'—C8'	118.0 (2)
C10—C9—C11	120.0 (3)	C5—C5'—C4'	122.2 (3)
C8'—C9—C11	120.4 (2)	C8'—C5'—C4'	119.8 (2)
C9—C10—C10'	122.9 (3)	C6—C5—C5'	122.0 (3)
C9—C10—H10	118.6	C6—C5—H5	119.0
C10'—C10—H10	118.6	C5'—C5—H5	119.0
C1—C10'—C4'	119.8 (3)	C5—C6—C7	119.9 (3)
C1—C10'—C10	120.9 (3)	C5—C6—H6	120.1
C4'—C10'—C10	119.2 (2)	C7—C6—H6	120.1
C2—C1—C10'	120.6 (3)	C8—C7—C6	120.3 (3)
C2—C1—H1	119.7	C8—C7—H7	119.8
C10'—C1—H1	119.7	C6—C7—H7	119.8
C1—C2—C3	119.9 (3)	C7—C8—C8'	121.2 (3)
C1—C2—H2	120.1	C7—C8—H8	119.4
C3—C2—H2	120.1	C8'—C8—H8	119.4
C4—C3—C2	120.9 (3)	C8—C8'—C5'	118.6 (3)
C4—C3—H3	119.5	C8—C8'—C9	122.0 (3)
C2—C3—H3	119.5	C5'—C8'—C9	119.3 (2)
C3—C4—C4'	120.9 (3)		
			
O1—N1—C11—C9	−175.3 (2)	C4—C4'—C5'—C5	−1.9 (4)
N1—C11—C9—C10	27.6 (4)	C10'—C4'—C5'—C8'	−0.5 (4)
N1—C11—C9—C8'	−156.1 (2)	C4—C4'—C5'—C8'	179.7 (3)
C8'—C9—C10—C10'	−0.2 (4)	C8'—C5'—C5—C6	0.2 (4)
C11—C9—C10—C10'	176.1 (3)	C4'—C5'—C5—C6	−178.2 (3)
C9—C10—C10'—C1	−179.3 (3)	C5'—C5—C6—C7	−0.1 (5)
C9—C10—C10'—C4'	−1.9 (4)	C5—C6—C7—C8	−0.1 (5)
C4'—C10'—C1—C2	0.4 (5)	C6—C7—C8—C8'	0.3 (5)
C10—C10'—C1—C2	177.9 (3)	C7—C8—C8'—C5'	−0.2 (4)
C10'—C1—C2—C3	0.0 (5)	C7—C8—C8'—C9	179.7 (3)
C1—C2—C3—C4	−0.2 (5)	C5—C5'—C8'—C8	−0.1 (4)
C2—C3—C4—C4'	0.0 (5)	C4'—C5'—C8'—C8	178.4 (3)
C1—C10'—C4'—C4	−0.5 (4)	C5—C5'—C8'—C9	−179.9 (2)
C10—C10'—C4'—C4	−178.1 (3)	C4'—C5'—C8'—C9	−1.5 (4)
C1—C10'—C4'—C5'	179.7 (3)	C10—C9—C8'—C8	−178.0 (3)
C10—C10'—C4'—C5'	2.2 (4)	C11—C9—C8'—C8	5.7 (4)
C3—C4—C4'—C10'	0.3 (4)	C10—C9—C8'—C5'	1.9 (4)
C3—C4—C4'—C5'	−179.9 (3)	C11—C9—C8'—C5'	−174.4 (2)
C10'—C4'—C5'—C5	177.8 (3)		

### (E)-Phenanthrene-9-carbaldehyde oxime (II) Hydrogen-bond geometry (Å, º)

**Table:**

*D*—H···*A*	*D*—H	H···*A*	*D*···*A*	*D*—H···*A*
O1—H···N1^i^	0.88 (3)	1.99 (3)	2.852 (3)	169 (3)

Symmetry code: (i) −*x*+1, *y*+1/2, −*z*+1.

## References

[bb1] Asaad, N., Davies, J. E., Hodgson, D. R. W., Kirby, A. J., van Vliet, L. & Ottavi, L. (2005). *J. Phys. Org. Chem.* **18**, 101–109.

[bb2] Bruker (2016). *APEX3*, *SAINT* and *SADABS*. Bruker AXS Inc., Madison, Wisconsin, USA.

[bb3] Bruton, E. A., Brammer, L., Pigge, F. C., Aakeröy, C. B. & Leinen, D. S. (2003). *New J. Chem.* **27**, 1084–1094.

[bb4] Groom, C. R., Bruno, I. J., Lightfoot, M. P. & Ward, S. C. (2016). *Acta Cryst.* B**72**, 171–179. 10.1107/S2052520616003954 PMC4822653 27048719

[bb5] Guo, Z., Li, L., Liu, G. & Dong, J. (2008). *Acta Cryst.* E**64**, o568. 10.1107/S160053680800370X PMC2960832 21201911

[bb6] Kassa, J. (2002). *J. Toxicol. Clin. Toxicol.* **40**, 803–816. 10.1081/clt-120015840 12475193

[bb7] Kopylovich, M. N., Haukka, M., Kirillov, A. M., Kukushkin, V. Yu. & Pombeiro, A. J. L. (2007). *Chem. Eur. J.* **13**, 786–791. 10.1002/chem.200600765 17048284

[bb8] Kopylovich, M. N., Kukushkin, V. Yu., Guedes da Silva, M. F. C., Haukka, M., Fraústo da Silva, J. J. R. & Pombeiro, A. J. L. (2001). *J. Chem. Soc. Perkin Trans. 1*, pp. 1569–1573.

[bb9] Kopylovich, M. N., Kukushkin, V. Yu., Haukka, M., Fraústo da Silva, J. J. R. & Pombeiro, A. J. L. (2002). *Inorg. Chem.* **41**, 4798–4804. 10.1021/ic0256720 12206707

[bb10] Kopylovich, M. N., Kukushkin, V. Yu., Haukka, M., Luzyanin, K. V. & Pombeiro, A. J. L. (2004). *J. Am. Chem. Soc.* **126**, 15040–15041. 10.1021/ja046759i 15547996

[bb11] Kopylovich, M. N., Lasri, J., Guedes da Silva, M. F. C. & Pombeiro, A. J. L. (2009). *Dalton Trans.* pp. 3074–3084. 10.1039/b820680e 19352536

[bb12] Kukushkin, V. Yu. & Pombeiro, A. J. L. (1999). *Coord. Chem. Rev.* **181**, 147–175.

[bb13] Lasri, J., Charmier, M. A. J., da Silva, M. F. C. G. & Pombeiro, A. J. L. (2007). *Dalton Trans.* pp. 3259–3266. 10.1039/b704329e 17893771

[bb14] Lasri, J., Guedes da Silva, M. F. C., Charmier, M. A. J. & Pombeiro, A. J. L. (2008). *Eur. J. Inorg. Chem.* pp. 3668–3677.

[bb15] Ritz, J., Fuchs, H., Kieczka, H. & Moran, W. C. (2012). *Ullmann’s Encyclopedia of Industrial Chemistry*. Weinheim: Wiley-VCH.

[bb16] Sheldrick, G. M. (2015*a*). *Acta Cryst.* A**71**, 3–8.

[bb17] Sheldrick, G. M. (2015*b*). *Acta Cryst.* C**71**, 3–8.

[bb18] Tarai, A. & Baruah, J. B. (2016). *Cryst. Growth Des.* **16**, 126–135.

